# Peak systolic longitudinal rotation: a new tool for detecting left ventricular systolic function in patients with type 2 diabetes mellitus by two-dimensional speckle tracking echocardiography

**DOI:** 10.1186/s12872-019-1119-y

**Published:** 2019-06-07

**Authors:** Jun Huang, Hai-Ling Hu, Zi-Ning Yan, Li Fan, Yi-Fei Rui, Dan Shen, Jie Li

**Affiliations:** 1 Department of Echocardiography, The Affiliated Changzhou No.2 People′s Hospital with Nanjing Medical University, Changzhou, 213003 China; 2 Department of Cardiology, Weifang Traditional Chinese Hospital, Weifang, 261046 China

**Keywords:** Type 2 diabetes mellitus, Longitudinal rotation, Two-dimensional speckle tracking echocardiography, Left ventricle, function

## Abstract

**Background:**

Type 2 diabetes mellitus (T2DM) is one of the most prevalent cardiac and cerebrovascular risk factors. The study aimed to find a new way to investigate left ventricle (LV) systolic dysfunction in T2DM patients using two-dimensional speckle tracking echocardiography (2D-STE).

**Methods:**

Fifty-one untreated T2DM patients and 52 normal control subjects were enrolled for the research. Apical four-chamber view was acquired by two-dimensional echocardiography. Segmental and global peak systolic longitudinal rotation (PSLR) degrees were measured by the software of EchoPAC.

**Results:**

In T2DM patients, global PSLR prominently rotated clockwise, while in normal subjects, global PSLR degrees were so small and almost had no PSLR. HBA1c negatively correlated with apex and global PSLR, that is, T2DM patients with higher HBA1c had a larger clockwise apex and global PSLR. ROC analysis showed that PSLR could detect the accuracy of LV systolic dysfunction.

**Conclusion:**

Cardiac clockwise global PSLR was found in T2DM patients. The cardiac contractile function in T2DM patients was impaired. The new tool of PSLR can conveniently detect cardiac systolic dysfunction in T2DM patients. HBA1c could predict systolic dysfunction in T2DM patients.

## Background

Type 2 diabetes mellitus (T2DM) is one of the most prevalent cardiac and cerebrovascular risk factors [1]. T2DM may alter cardiac structure and function independently of underlying coronary artery disease or hypertension [2]. Left ventricle (LV) remodeling may lead to diabetic cardiomyopathy before LV contractile dysfunction [3]. Initial assessment of LV dysfunction in T2DM patients is vital for the treatment and prognosis.

Two-dimensional speckle tracking echocardiography (2D-STE) has been introduced as a novel method for angle-independent and well reproducibility quantification of LV strain, strain rate and LV twist [4–7], and by the measurement of these values, LV dysfunction could be assessed. The speckles are the result of constructive and destructive interference of conventional gray scale ultrasound images.

Most of previous studies have measured LV strain, strain rate, twist or torsion by 2D-STE or 3D-STE in T2DM patients for detecting LV systolic and diastole dysfunction, and then demonstrated the impairment of LV function [8–12].

Peak systolic longitudinal rotation (PSLR) as a novel marker means the rotational motion in the long axis of heart. Previous studies have demonstrated that PSLR were small in normal subjects, however, systolic clockwise PSLR motion have been detected in dilated cardiomyopathy, hypertrophic cardiomyopathy, ischemic cardiomyopathy, and arterial hypertension patients [13–15], however, the etiology of PSLR is still unknown.

In the research, we want to find a simple method to evaluate the LV contractile dysfunction in T2DM patients. We phased a specific hypothesis that T2DM patients had PSLR in cardiac cycle. The importance and necessity of the study was to determine whether T2DM patients had cardiac PSLR, and evaluated the relationship between PSLR and clinical laboratory indicators, just like fasting plasma glucose and HBA1c. At last, provide a simple and accurate method to detect LV systolic dysfunction in T2DM patients.

## Methods

### Ethical statement

The Human Subjects Committee of the Affiliated Changzhou No.2 People′s Hospital with Nanjing Medical University approved this study. Written informed consent was obtained from each patient enrolled to the research. The reference number for the ethical approval is: [2014]KY010–01.

### Study population

**Fig. 1:**
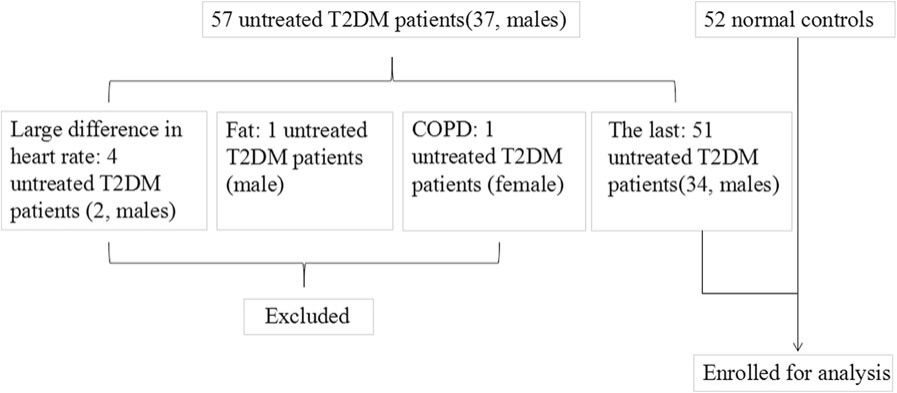
Flow diagram showed the justification and inclusion/exclusion criteria of T2DM patients and normal controls

Fifthy-seven untreated T2DM patients (37, males) and 52 normal controls (27, males) of similar age and gender were enrolled for this study, however, 4 untreated T2DM patients (2, males) were excluded for the large difference in heart rate, 1 untreated T2DM patients (male) was excluded for fat, and 1 untreated T2DM patients (female) was excluded for COPD. At last, 51 untreated T2DM patients (34, males) were enrolled for the study (Fig. 1). This is a cross-sectional study that was conducted between August 2015 and May 2017, and data was collected prospectively. Subjects with cardiovascular disease, such as coronary artery disease (all of the patients were had coronary artery CT scan to ensure that they had no coronary artery disease), arterial hypertension, myocardial infarction, cardiomyopathy, valvular disease, atrial fibrillation, congenital heart disease, thyroid disease, neoplastic disease, or kidney failure were excluded from the study. In normal control subjects, all of the physical examination tests, the electrocardiogram, and the echocardiography were showed normal. All normal subjects had an absence of diabetes (according to the diagnosis of T2DM, the fasting blood glucose, Two-hour postprandial blood sugar and HbA1c were all showed normal).

### Biochemistry

In T2DM patients and normal subjects, fasting plasma glucose, two-hour postprandial blood sugar and glycated hemoglobin (HBA1c) were measured.

### Conventional 2D Doppler echocardiography

All enrolled subjects underwent conventional 2D Doppler echocardiography (Vivid E9, GE healthcare). Echocardiography examination was done before beginning drugs in T2DM patients. Left atrial diameter (LAD), interventricular septum thickness and LV posterior wall thickness in end-diastole (IVSD and LVPWD) were measured in the parasternal long-axis view of LV by M-mode. LV end-diastole volume (LVEDV), LV end-systole volume (LVESV) and LV ejection fraction (LVEF) were measured by modified biplane Simpson′s method. Peak early and late diastolic velocities of mitral valve (E and A, respectively) were measured by pulsed-wave Doppler, and the ratio of E/A was then calculated. Peak early (e′) and late (a′) diastolic annular velocities were obtained by averaging the values at septum and lateral positions using pulse wave Tissue Doppler Imaging (TDI), and E/e′ was calculated.

ECG leads were connected to each patient. Standard high frame rate (> 60 /s) of the apical four-, three- and two-chamber views of three consecutive cycles while patients held their breath were stored for offline analysis.

### Analysis of LV systolic function

The apical four-, three- and two-chamber views were analyzed using 2D-STE software (2D-Strain, EchoPAC PC 113, GE Healthcare, Horten, Norway) by two experienced cardiologist.

**Fig. 2:**
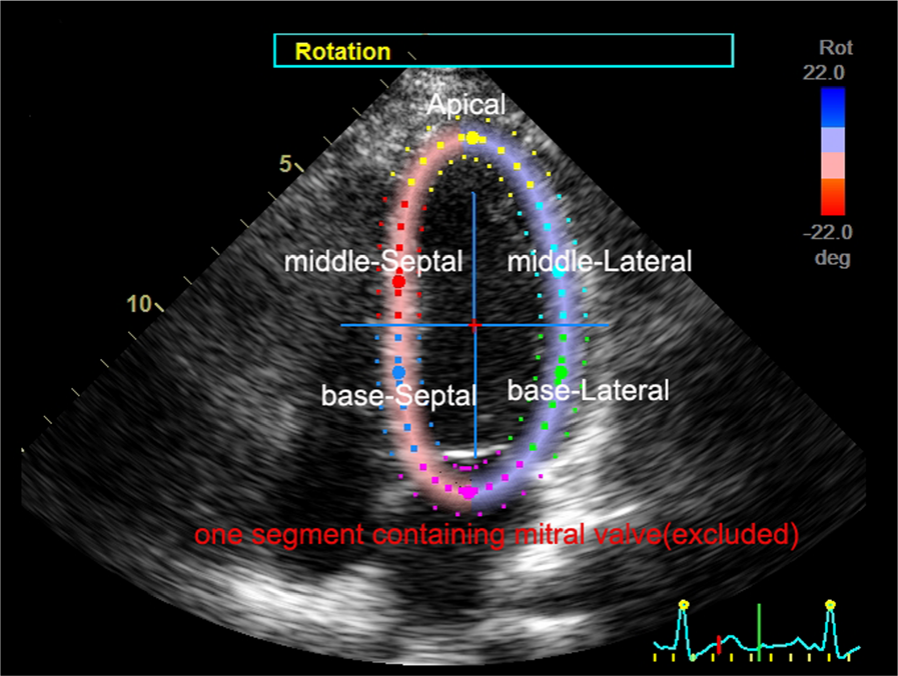
The measurement of PSLR in T2DM patients and normal subjects

First, we defined cardiac PSLR as the rotation of the LV cross section. Segmental and global PSLR degrees were measured. Using the SAX-MV option of EchoPAC software displayed on the apical four-chamber view. The LV walls were divided into six segments: base-septal, middle-septal, apex, middle-lateral, and base-lateral, and one segment containing mitral valve, respectively. The segment containing mitral valve was excluded for the analysis. Then segmental and global PSLR of LV walls were measured via the software (Fig. 2).

Second, we used LAX, A4C and A2C options for the analysis of LV longitudinal strain and strain rate by the software, “LAX” means apical three chamber view. LV longitudinal strain (including LS-endo, LS-mid, and LS-epi, which represented LV endomycardial, middle myocardial and epimycardial walls, respectively) and LV longitudinal strain rate (LSr) were calculated and recorded.

### Statistical analysis

All data analyses were performed using SPSS 21.0 software (SPSS, Chicago, IL, USA). Data was presented as the mean ± standard deviation (SD). *p*-value < 0.05 was considered statistically significant in all tests. Kolmogorov-Smirnov′s test was used to detect the normality of all the segmental and global PSLR values. Differences between T2DM patients and normal subjects were compared with an independent Student′s t-test for the data distribution was normal. Differences among the first analysis, interobserver and intraobserver were compared with one-way analysis of variance (ANOVA). For variables with a non-normal distribution, the nonparametric U Mann-Whitney test was used. Chi square was used for comparing the variable of sex. Spearman′s correlation was chosen for the test correlations among the fasting plasma glucose, HBA1c, LVEF, LS-endo, LS-mid, LS-epi, LSr, segmental PSLR and global PSLR. We defined the apex and global PSLR in control subjects as the normal state, and considered the values of T2DM patients as abnormal. Values for apex and global PSLR in T2DM patients were determined from receiver operating characteristic (ROC) curve analysis. Yoden′s index was selected for the cut-off point which can give the best composite of specificity and sensitivity.

### Reproducibility and repeatability

Intraobserver and interobserver variability for global PSLR were determined by repeating measurements in all enrolled T2DM patients and normal subjects. For the second intraobserver measurements, the observer was “blinded” to results of the initial measurements.

## Results

### Basic information in T2DM patients and normal subjects

There were significant differences in body weight, BSA (body surface area), BMI (body mass index), HR, SBP, DBP, LVEDV, Indexed LVEDV, indexed LVESV, LVEF, e′, a′ and E/E′. LVEDV, indexed LVEDV, indexed LVESV, LVEF and e′ in T2DM patients were significantly lower than normal subject, while a′ and E/e′ were significantly larger than normal subjects. (Table 1).

**Table 1:** Baseline clinical characteristics, conventional two-dimensional echocardiographic parameters between T2DM patients and normal subjects (mean ± SD)

Variable		T2DM	Normal	P
Clinical	Age (yrs)	55.57 ± 11.08	50.42 ± 13.88	0.207
	Male	34(51)	27(52)	0.128
	Height(cm)	166.08 ± 8.64	163.60 ± 8.15	0.137
	Weight(kg)	66.08 ± 11.61	56.68 ± 8.57	**< 0.001**
	BSA(m^2^)	1.71 ± 0.19	1.57 ± 0.14	**< 0.001**
	BMI (kg/m^2^)	22.67 ± 1.44	21.99 ± 1.18	0.010
	Heart Rate(bpm)	76.39 ± 9.52	71.85 ± 11.49	0.031
	SBP (mmHg)	127.55 ± 12.64	118.25 ± 10.36	**< 0.001**
	DBP (mmHg)	78.10 ± 8.85	72.60 ± 7.54	**0.001**
	Fasting plasma glucose (mmol/L)	13.41 ± 4.38	4.82 ± 0.64	**< 0.001**
	Two-hour postprandial blood sugar(mmol/L)	15.13 ± 4.52	5.66 ± 0.73	**< 0.001**
	HbA1c (%)	10.34 ± 2.25	4.98 ± 0.73	**< 0.001**
	NYHA Class			
	I	51(51)	52(52)	
	II	0(51)	0(52)	
	III	0(51)	0(52)	
	IV	0(51)	0(52)	
	Medical treatment			
	Diet treatment	0(51)		
	Oral drug	10(51)		
	Insulin	26(51)		
	Insulin+Oral drug	15(51)		
Echocardiography	LA D(mm)	35.53 ± 3.88	34.65 ± 3.29	0.219
	IVSD(mm)	9.39 ± 1.25	9.15 ± 0.98	0.283
	LVPWD(mm)	9.06 ± 1.21	9.00 ± 1.08	0.795
	LVDD(mm)	47.00 ± 3.64	46.94 ± 3.15	0.932
	LVEDV(ml)	72.29 ± 14.78	79.02 ± 12.64	**0.015**
	Indexed LVEDV (ml/m^2^)	42.72 ± 9.25	50.63 ± 8.68	**< 0.001**
	LVESV(ml)	27.39 ± 5.99	28.29 ± 7.61	0.509
	Indexed LVESV (ml/m^2^)	16.16 ± 3.58	18.08 ± 4.80	0.023
	LVEF(%)	62.06 ± 4.75	64.53 ± 5.51	**0.017**
	LV mass(g)	149.81 ± 34.70	145.92 ± 31.07	**0.550**
	Indexed LV mass(g/m^2^)	88.49 ± 21.71	92.95 ± 18.42	**0.263**
	E(m/s)	0.79 ± 0.14	0.83 ± 0.16	0.296
	A(m/s)	0.69 ± 0.19	0.69 ± 0.18	0.958
	E/A	1.23 ± 0.35	1.27 ± 0.38	0.590
	e′(m/s)	0.09 ± 0.02	0.11 ± 0.02	**0.001**
	a′ (m/s)	0.10 ± 0.02	0.08 ± 0.02	**< 0.001**
	E/e′	10.39 ± 2.50	8.13 ± 2.61	**< 0.001**
Speckle Tracking Echocardiography	LS-endo	−23.46 ± 2.42	−24.22 ± 2.99	0.160
	LS-mid	−20.29 ± 2.15	−20.92 ± 2.61	0.183
	LS-epi	−17.64 ± 1.94	−18.15 ± 2.35	0.237
	LSr	−1.06 ± 0.16	−1.12 ± 0.19	0.088

BSA: Body surface area, BMI: Body mass index, SBP: systolic blood pressure, DBP: diastolic blood pressure, LAD: left atrial diameter, IVSD: interventricular septal thickness in end-diastolic period, LVPWD: left ventricular posterior wall thickness in end-diastolic period, LVDD: left ventricular diameter in end-diastolic period, LVEDV: left ventricular end-diastolic volume, LVESV: left ventricular end-systolic volume, LVEF: left ventricular ejection fraction, E: peak velocity during early diastole of anterior mitral valve, A: peak velocity during late diastole of anterior mitral valve, e′: peak early diastolic annular velocities using TDI, a′: peak late diastolic annular velocities using TDI. LS-endo: longitudinal strain of LV endomyocardial. LS-mid: longitudinal strain of LV middle myocardial. LS-epi: longitudinal strain of LV epimyocardial. LSr: longitudinal strain rate of LV

### Segmental and global PSLR

**Fig. 3:**
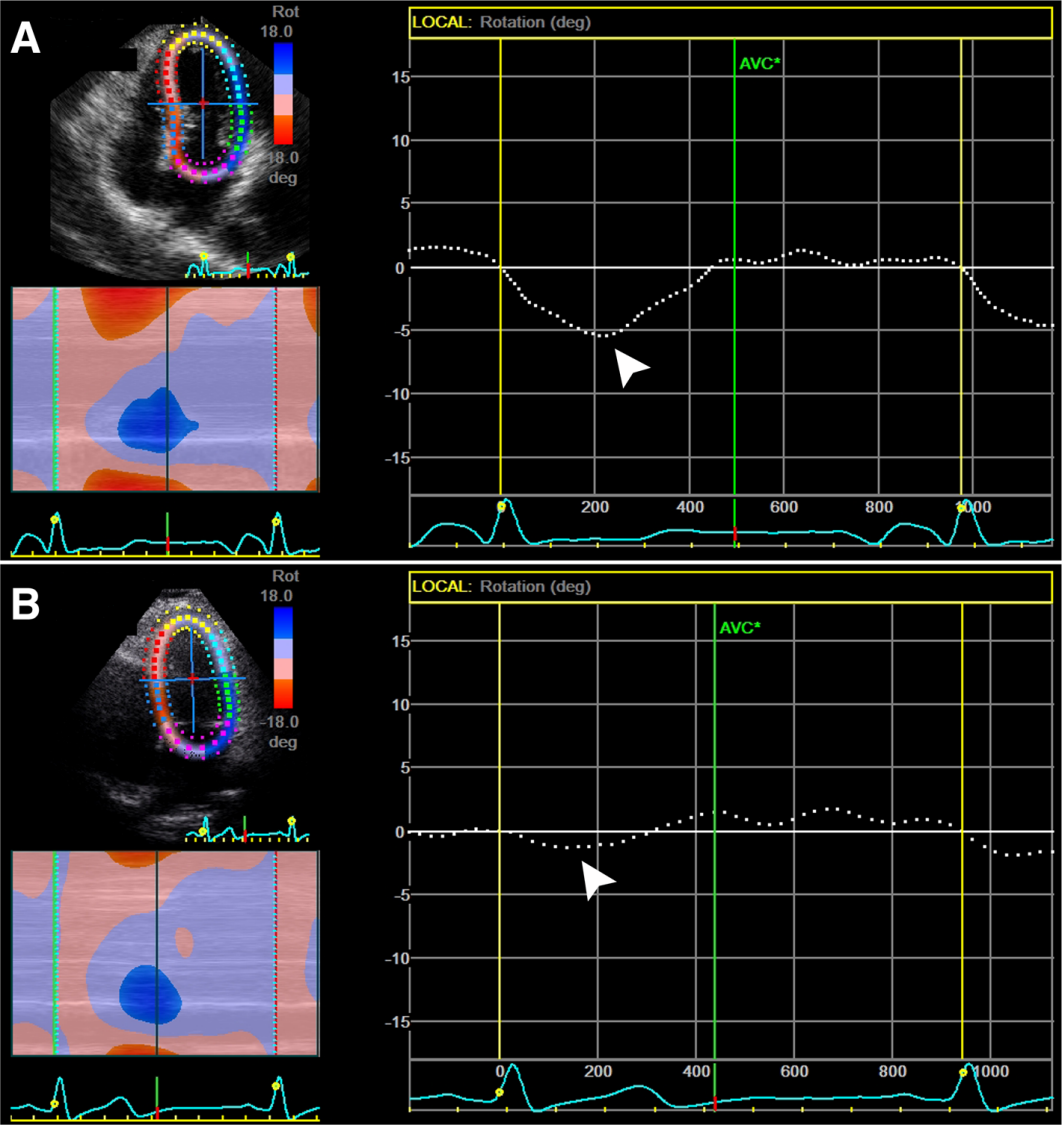
Global PSLR in T2DM patients (A) and normal subjects (B). PSLR: Peak systolic longitudinal rotation

In T2DM and normal subjects, segmental PSLR of lateral wall rotated counter-clockwise, while septum rotated clockwise. In normal subjects, global PSLR degrees were so small and almost had no PSLR. In T2DM patients, global PSLR prominently rotated clockwise. A smaller PSLR of base-lateral, middle-lateral walls, while a larger apex and global PSLR in T2DM patients were observed when compared with normal subjects. There were no significant differences in base-septum and middle septum between the two groups. (Table 2, Fig. 3).

**Table 2:** Segmental and global peak systolic longitudinal rotation (PSLR) between T2DM patients and normal subjects (mean ± SD)

Variable		T2DM	Normal	P
PSLR	base-Lateral	7.91 ± 4.24	9.86 ± 3.44	**0.012**
	middle-Lateral	4.90 ± 4.32	6.49 ± 3.66	**0.046**
	Apex	−1.00 ± 3.95	0.86 ± 3.60	**0.014**
	middle-Septal	−6.72 ± 3.03	−5.21 ± 4.87	0.062
	base-Septal	−9.53 ± 2.31	−10.05 ± 3.12	0.340
	Global	−2.18 ± 3.26	−0.18 ± 2.50	**0.001**

### Correlations among fasting plasma glucose, HBA1c, LVEF, LS-endo, LS-mid, LS-epi, LSr with the segmental and global PSLR in T2DM patients

**Fig. 4:**
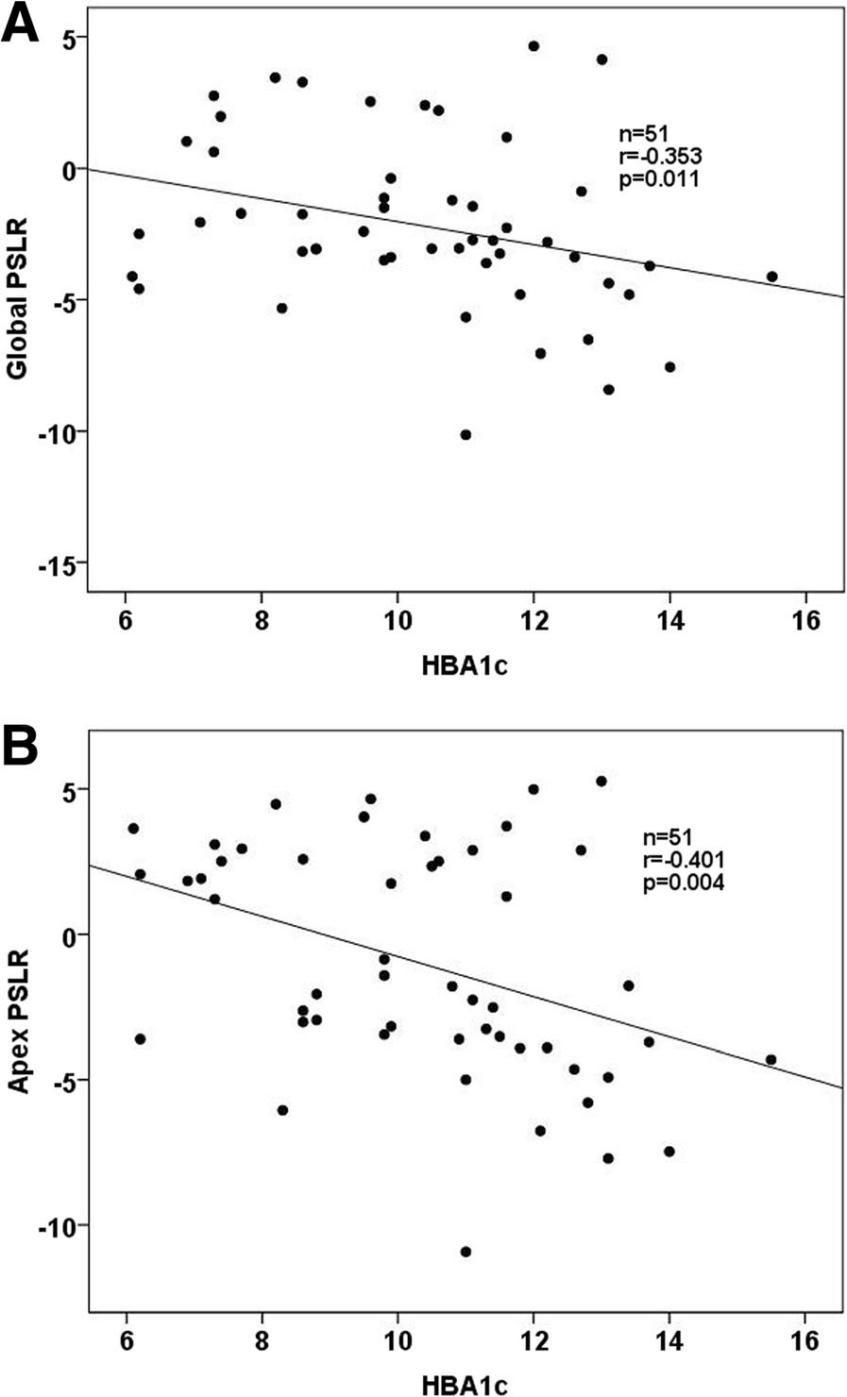
Correlation between global PSLR and HBA1c in T2DM patients (**a**). Correlation between apex PSLR and HBA1c in T2DM patients (**b**). Global and apex PSLR negatively correlated with HBA1c in T2DM patients

HBA1c negatively correlated with apex and global PSLR (r = − 0.401, *p* = 0.004, and r = − 0.353, *p* = 0.001), respectively, that is, T2DM patients with higher HBA1c had a larger clockwise apex and global PSLR. LSr negatively correlated with PSLR of lateral wall. There were no correlations among fasting plasma glucose, LVEF, LS-endo, LS-mid, LS-epi with the segmental and global PSLR. (Table 3, Fig. 4).

**Table 3:** Correlations between segmental PSLR, global PSLR and GLU, HBA1c and LVEF in T2DM patients

Variable	Fasting plasma glucose		HBA1c		LVEF		LS-endo		LS-mid		LS-epi		LSr	
	r	*p*-value	r	*p*-value	r	*p*-value	r	*p*-value	r	*p*-value	r	*p*-value	r	*p*-value
base-Lateral	−0.156	0.273	−0.018	0.898	0.127	0.375	−0.082	0.567	−0.103	0.470	−0.145	0.312	−0.351	**0.011**
middle-Lateral	−0.213	0.134	−0.062	0.664	−0.017	0.904	−0.138	0.333	−0.137	0.337	−0.157	0.270	−0.447	**0.001**
Apex	−0.263	0.062	**−0.401**	**0.004**	−0.017	0.904	0.135	0.346	0.103	0.474	0.067	0.643	−0.147	0.304
middle-Septal	−0.141	0.325	−0.226	0.111	−0.018	0.901	0.251	0.076	0.221	0.119	0.170	0.232	0.019	0.893
base-Septal	−0.028	0.847	−0.270	0.055	0.000	0.996	0.203	0.154	0.205	0.149	0.188	0.187	0.059	0.679
Global	−0.196	0.168	**−0.353**	**0.011**	−0.087	0.546	0.214	0.132	0.188	0.187	0.144	0.313	−0.093	0.517

### ROC analysis for detecting the accuracy of apex and global PSLR

**Fig. 5:**
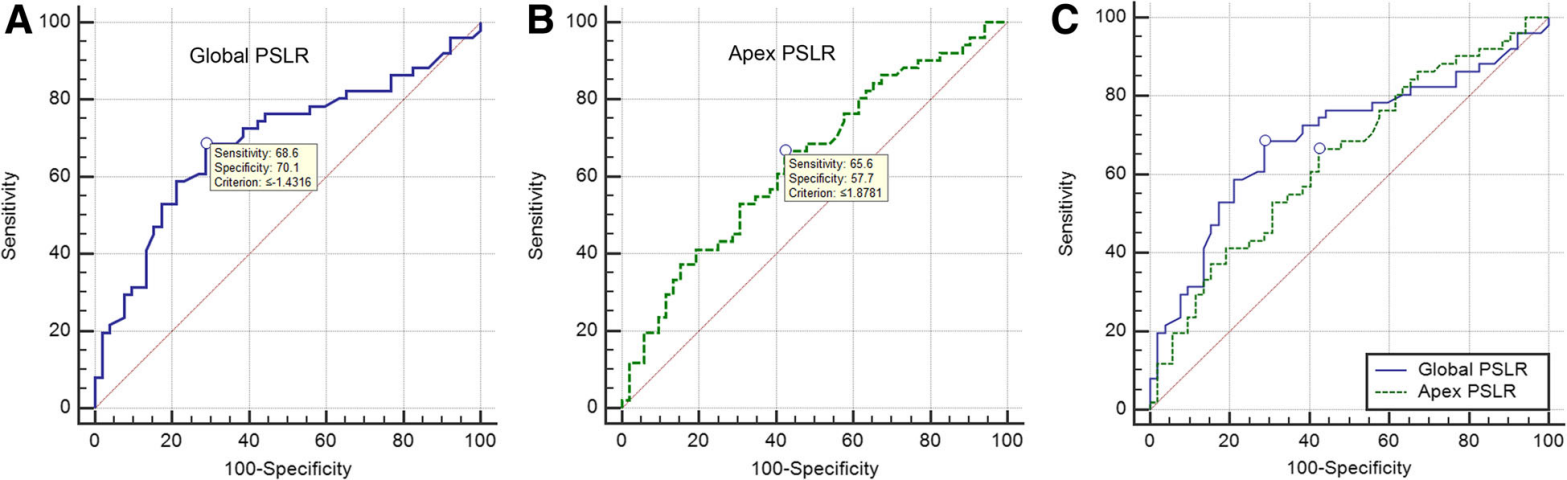
ROC analysis for detecting the accuracy of apex and global PSLR. The area under ROC curve values, sensitivity, specificity, cut-off value and Youden index for apex PSLR in DM patients were 0.693, 68.63, 71.15%, − 1.45 and 0.3978, respectively (**a**). The area under ROC curve values, sensitivity, specificity, cut-off value and Youden index for global PSLR in DM patients were 0.641, 66.67, 57.79%, 1.92 and 0.2436, respectively (**b**). Comparison of ROC analysis curves between apex and global PSLR had no significant difference (**c**)

The area under ROC curve values, sensitivity, specificity, cut-off value and Youden index for apex PSLR in T2DM patients were 0.693, 68.63, 71.15%, − 1.45 and 0.3978, respectively. The area under ROC curve values, sensitivity, specificity, cut-off value and Youden index for global PSLR in T2DM patients were 0.641, 66.67, 57.79%, 1.92 and 0.2436, respectively. Comparison of ROC analysis curves between apex and global PSLR showed no significant difference (*p* = 0.189). (Table 4, Fig. 5).

**Table 4:** ROC analysis for detecting the accuracy of apex and global PSLR in T2DM patients

Variable	Global LR	Apex LR	*p*-value
Sensitivity	68.63	66.67	0.189
Specificity	71.15	57.69	
Cut-off Value	−1.45	1.92	
Area under curve	0.693	0.641	
Youden index	0.3978	0.2436

### Reproducibility and repeatability

The results for the intraobserver and interobserver variability for the global PSLR upon repeated measurements in all study patients were shown in Table 5. We compared the interobserver and intraobserver variability with the first time, and found there were no differences among the three time. There were significant differences between T2DM patients and normal subjects in interobserver and intraobserver analysis. What′s more, the results demonstrated that the analysis of the software had the well repeatability and reliability of the study.

**Table 5:** Interobserver and intraobserver reproducibility and repeatability

Variable	Global PSLR(°)						*P*-value
	First analysis		Interobserver		Intraobserver		
	Mean ± SD	95% CI	Mean ± SD	95% CI	Mean ± SD	95% CI	
T2DM	−2.18 ± 3.26	−3.10~ −1.26	−2.16 ± 3.21	−3.06~ − 1.26	−2.25 ± 3.27	−3.17~ − 1.33	0.989
Normal	−0.18 ± 2.50	−0.87~0.52	−0.16 ± 2.42	− 0.89~ 0.47	−0.17 ± 2.39	− 0.90~ 0.45	0.999
*P*-value	0.001		0.001		< 0.001		

## Discussion

The main findings of the research were: ①Global PSLR motion prominently rotated clockwise in T2DM patients. ②Higher HBA1c had larger apex and global PSLR in T2DM patients. ③Apex and global PSLR could reflect LV contractile dysfunction in T2DM patients. No significant difference was found between the two results.

LVEF is always used for detecting contractile function in normal subjects and cardiac disease patients. However, in the study, LVEF was similar in T2DM patients and normal subjects. It cannot reflect early systolic dysfunction in T2DM patients. Previous reports used 2D-STE or 3D-STE to measure strain, strain rate or LV torsion for assessment of cardiac systolic and diastolic dysfunction. Ernande L et al. [16] used 2D-STE to measure longitudinal and radial systolic strain in T2DM patients, and found that longitudinal and radial systolic function were impaired in asymptomatic patients with T2DM. Enomoto M et al. [17] found global longitudinal and circumferential strain were lower in patients with T2DM than in the controls by using 3D-STE, and concluded that diabetic microangiopathy and its accumulated effects significantly related to subclinical LV dysfunction were characterized by impaired longitudinal shortening.

In the research, we introduced a new way to detect systolic dysfunction which was defined as PSLR. PSLR was first reported by Popović ZB in 2007, and the researcher found that in dilated cardiomyopathy and ischemic cardiomyopathy patients, the cardiac had LR [13]. The orientation of the LR is unclear at present. In T2DM patients, prominently clockwise PSLR was detected. A smaller PSLR of base-lateral, middle-lateral walls, while a larger apex and global PSLR in T2DM patients was observed when compared with normal subjects, and there was no significant difference in septum wall. The pathogenesis of T2DM cardiomyopathy is likely to be multifactorial, including micro vascular disease, altered myocardial metabolism, and structural changes in the myocardium with increased fibrosis [18]. We considered the orientation of PSLR may be linked to myocardium fibrosis. According to the theory of “myocardial band” [19], and a normal myocardium consists of subendocardial, middle, and subepicardial fibers. When the normal myocardium became fibrosis, we thought the primary balance among the myocardium was disappeared, as a result, PSLR was detected. Further researches of our group want to verify the hypothesis between PSLR and myocardium fibrosis in T2DM animal model.

From the results, we found that there were no differences in LS-endo, LS-middle, LS-epi and LSr between T2DM patients and normal subjects, however, global, apex and lateral PSLR had the significant difference between the two groups, so we concluded that PSLR was an accurate and simple method.

HBA1c negatively correlated with apex and global PSLR, respectively. No correlations were discovered among fasting plasma glucose, LVEF with segmental and global PSLR. Therefore, higher HBA1c of T2DM patients had larger apex and global PSLR. ROC analysis showed that the area under ROC curve values of apex and global PSLR was: 0.693 and 0.641. Comparison of ROC analysis curves between apex and global LR had no significant difference. From the results, we concluded that apex and global PSLR could reflect the contractile dysfunction in T2DM patients. HBA1c could predict systolic dysfunction in T2DM patients.

## Conclusions

In the study, we found contractile function was impaired in T2DM patients. Cardiac clockwise PSLR was found in T2DM patients. The new tool PSLR can be flexible and conveniently to detect cardiac systolic dysfunction in T2DM patients. HBA1c could also predict systolic dysfunction in T2DM patients.

### Limitations

First, the number of T2DM patients and normal subjects in this study was small. Second, the technique of EchoPAC in the analysis of two-dimensional images has the shortcoming of out-of plane motion, if the speckles move out of plane during contraction, the software cannot be tracked successfully. In the research, we just told the patients hold their breath to prevent the condition. Third, large differences in heart rate, fat patients and patients with COPD were insufficient and excluded for the analysis.

## Data Availability

The datasets used and/or analysed during the current study available from the corresponding author on reasonable request.

## Abbreviations

2D-STE: Two-dimensional speckle tracking echocardiography
IVSD: Interventricular septal thickness in end-diastolic period
LAD: Left atrial diameter
LV: Left ventricle/ventricular
LVEDV: Left ventricular end-diastolic volume
LVEF: Left ventricular ejection fraction
LVESV: Left ventricular end-systolic volume
LVPWD: LV posterior wall thickness in end-diastolic period
PSLR: Peak systolic longitudinal rotation
T2DM: Type 2 diabetes mellitus
TDI: Tissue Doppler imaging

## References

[1] Tadic M, Ilic S, Cuspidi C, Stojcevski B, Ivanovic B, Bukarica L (2015). Left ventricular mechanics in untreated normotensive patients with type 2 diabetes mellitus: a two- and three-dimensional speckle tracking study. Echocardiography..

[2] Philouze C, Obert P, Nottin S, Benamor A, Barthez O, Aboukhoudir F (2018). Dobutamine stress echocardiography unmasks early left ventricular dysfunction in asymptomatic patients with uncomplicated type 2 diabetes: a comprehensive two-dimensional speckle-tracking imaging study. J Am Soc Echocardiogr.

[3] Guo R, Wang K, Song W, Cong T, Shang ZJ, Sun YH (2014). Myocardial dysfunction in early diabetes patients with microalbuminuria: a 2-dimensional speckle tracking strain study. Cell Biochem Biophys.

[4] Notomi Y, Lysyansky P, Setser RM, Shiota T, Popović ZB, Martin-Miklovic MG (2005). Measurement of ventricular torsion by two-dimensional ultrasound speckle tracking imaging. J Am Coll Cardiol.

[5] Helle-Valle T, Crosby J, Edvardsen T, Lyseggen E, Amundsen BH, Smith HJ (2005). New noninvasive method for assessment of left ventricular rotation: speckle tracking echocardiography. Circulation.

[6] Huang J, Yan ZN, Rui YF, Fan L, Shen D, Chen DL (2016). Left ventricular systolic function changes in primary hypertension patients detected by the strain of different myocardium layers. Medicine (Baltimore).

[7] Huang J, Yan ZN, Rui YF, Fan L, Liu C, Li J (2018). Left ventricular short-axis systolic function changes in patients with hypertrophic cardiomyopathy detected by two-dimensional speckle tracking imaging. BMC Cardiovasc Disord.

[8] Li RJ, Yang J, Yang Y, Ma N, Jiang B, Sun QW (2014). Speckle tracking echocardiography in the diagnosis of early left ventricular systolic dysfunction in type II diabetic mice. BMC Cardiovasc Disord.

[9] Wang Q, Tan K, Xia H, Gao Y (2018). Left ventricular structural alterations are accompanied by subclinical systolic dysfunction in type 2 diabetes mellitus patients with concomitant hyperlipidemia: an analysis based on 3D speckle tracking echocardiography. Echocardiography.

[10] Li ZJ, Du LF, Luo XH (2014). Evaluation of ventricular-vascular coupling in patients with type 2 diabetes mellitus using 2-dimensional speckle tracking imaging. J Huazhong Univ Sci Technolog Med Sci.

[11] Wang Q, Gao Y, Tan K, Xia H, Li P (2015). Assessment of left ventricular function by three-dimensional speckle-tracking echocardiography in well-treated type 2 diabetes patients with or without hypertension. J Clin Ultrasound.

[12] Wang Q, Ma W, Xia J (2018). Nonalcoholic fatty liver is associated with further left ventricular abnormalities in patients with type 2 diabetes mellitus: a 3-dimensional speckle-tracking study. J Ultrasound Med.

[13] Popović ZB, Grimm RA, Ahmad A, Agler D, Favia M, Dan G (2007). Longitudinal rotation: an unrecognised motion pattern in patients with dilated cardiomyopathy. Heart.

[14] Huang J, Yan ZN, Ni XD, Hu YP, Rui YF, Fan L (2016). Left ventricular longitudinal rotation changes in primary hypertension patients with normal left ventricular ejection fraction detected by two-dimensional speckle tracking imaging. J Hum Hypertens.

[15] Huang J, Yan ZN, Fan L, Rui YF, Song XT (2017). Left ventricular systolic function changes in hypertrophic cardiomyopathy patients detected by the strain of different myocardium layers and longitudinal rotation. BMC Cardiovasc Disord.

[16] Ernande L, Rietzschel ER, Bergerot C, De Buyzere ML, Schnell F, Groisne L (2010). Impaired myocardial radial function in asymptomatic patients with type 2 diabetes mellitus: a speckle-tracking imaging study. J Am Soc Echocardiogr.

[17] Enomoto M, Ishizu T, Seo Y, Kameda Y, Suzuki H, Shimano H (2016). Myocardial dysfunction identified by three-dimensional speckle tracking echocardiography in type 2 diabetes patients relates to complications of microangiopathy. J Cardiol.

[18] Ng AC, Delgado V, Bertini M, van der Meer RW, Rijzewijk LJ, Shanks M (2009). Findings from left ventricular strain and strain rate imaging in asymptomatic patients with type 2 diabetes mellitus. Am J Cardiol.

[19] Torrent-Guasp FF, Whimster WF, Redmann K (1997). A silicone rubber mould of the heart. Technol Health Carem.

